# Comparing Digital Reduction Guides and Free‐Hand Techniques for Predoctoral Crown Preparation Accuracy

**DOI:** 10.1002/jdd.70163

**Published:** 2026-02-10

**Authors:** Xiaoyue Shi, Shuncong Xu, Chenxuan Wei

**Affiliations:** ^1^ Department of Biologic and Materials Sciences & Prosthodontics School of Dentistry University of Michigan Ann Arbor Michigan USA; ^2^ HeyGears IMC.INC Guangzhou China

## Abstract

**Purpose/Objectives:**

Insufficient tooth reduction compromises crown longevity and aesthetics. This study aimed to determine whether digitally designed, 3D‐printed reduction guides improved crown preparation accuracy and efficiency for dental students compared to freehand preparation.

**Methods:**

In a randomized crossover, third‐ and fourth‐year students (*N* = 25) prepared typodont #8 twice—freehand and with a segmented guide (incisal, axial, and lingual components) designed per ADEX standards. Preparation quality was assessed by an independent, blinded examiner using an 11‐criteria rubric (Ideal/Substandard/Critical) and digital analysis (CEREC SW 5.2). Guide usability was examined with a NASA‐TLX survey.

**Result:**

The guided method significantly improved preparation accuracy scores by 1.12 points (*p* < 0.05) compared to freehand, with 64% of students achieving higher scores, and reduced preparation time by 17% (*p* < 0.05). Although 48% of participants rated the task difficulty as “Somewhat High” on the TLX subscale, 92% had a global NASA‐TLX score < 50 (below the “High” workload threshold), indicating that the overall perceived workload remained below the “High” category and that most users found the guide easy to use.

**Conclusion:**

The custom 3D‐printed reduction guide significantly improved both accuracy and efficiency of anterior crown preparation among dental students. These findings support the integration of digitally fabricated guides into preclinical curricula to standardize outcomes and bridge gaps between training and practice through digital workflows.

## 1 Introduction

Preclinical full crown preparation is a fundamental and challenging skill for dental students. Mastering the required tooth reduction using only free‐hand techniques often proves difficult for novices, resulting in inconsistent preparation quality [1]. Evaluation of student work by instructors can also be subjective and variable; one study reported only slight to fair agreement between faculty graders on key crown preparation criteria [2]. This variability in both student performance and assessment underscores the need for more standardized and effective teaching methods in prosthodontic training.

In response, digital innovations such as intraoral scanners and digital evaluation software now allow objective feedback on preparation dimensions, supplementing traditional teaching [3]. Video‐based digital evaluation improves competency in all‐ceramic crown preparation [4]. Recent studies [3, 5] demonstrate that digital‐conventional hybrid teaching methods outperform traditional approaches in skill retention and preparation consistency, yet few address guided tactile feedback for anterior teeth. Three‐dimensional (3D) printing enables custom reduction guides that direct tooth reduction. These 3D‐printed guides provide a standardized reference for ideal crown preparation form, potentially minimizing errors and inter‐student variability [6]. Clinical uses of preparation guides have also been reported, including pediatric zirconia crowns, supporting the feasibility of guided reduction in patient care [7]. Customized guides have also been used clinically to control anterior contour and limit reduction prior to minimally invasive preparations [8]. Early evidence indicates that inexperienced students using 3D‐printed reduction guides produce more accurate preparations [1], but most guides use monolithic designs with limited adaptability for anterior geometry. Our study addresses this gap by introducing a segmented, tri‐component design (incisal/axial/lingual), systematically standardizing reduction stages, and a feature underexplored in previous work.

The aim of this study was to examine whether a segmented 3D‐printed reduction guide can help students achieve more accurate and efficient anterior tooth preparations than the free‐hand approach in a predoctoral simulation setting. The workflow uses intraoral scanning and design software to create custom guides that support ideal tooth reduction in simulation. Accordingly, we tested the null hypothesis (H_0_) that use of the segmented 3D‐printed reduction guide results in no difference in mean preparation quality scores or preparation times relative to free‐hand preparation (two‐tailed *α* = 0.05).

## 2 Materials and Methods

### 2.1 Reduction Guide Design and Fabrication

A typodont model (ModuPRO One, model MP_R320, Acadental, Overland Park, KS, USA) was used to simulate the dental arch and tooth anatomy for guide development. Hand dominance was not recorded; all procedures were performed on a typodont mounted in a manikin head using the same simulation setup, and the guide's seating geometry (incisal/lingual stops) was identical for all participants. Initial guide prototypes were conceptualized and validated using traditional analog methods: a stone cast of the typodont tooth, silicone putty stents of an ideal preparation form, and a light‐cured resin material (Traid, Dentsply Sirona, Charlotte, NC, USA) to shape preliminary guide forms. The design was iteratively refined by accounting for the 1 mm non‐cutting tip present on the crown preparation bur, ensuring the guide would not facilitate cutting beyond a safe depth. The final guide design was created digitally using PlastyCAD software (1.7 version, 3DIEMME, Como, Italy), following the 2024 ADEX all‐ceramic anterior crown preparation standards for tooth reduction dimensions and geometry. The guiding system was segmented into three separate components corresponding to sequential cutting stages of the crown preparation. Each guide piece directed a specific phase of tooth reduction (e.g., incisal reduction, facial reduction, and lingual/palatal reduction) to progressively achieve the ideal preparation form. The guide components were manufactured with a stereolithography 3D printer (SprintRay Pro, SprintRay Inc., Los Angeles, CA, USA) using a biocompatible surgical guide resin, producing a transparent rigid guide that precisely fit over the crown of tooth #8. This segmented reduction‐guide concept is covered by a Chinese utility model patent (CN223009274U; filed July 26, 2024; granted June 24, 2025), on which our second author, Shuncong Xu, is a named inventor. Three prototype iterations were tested on typodonts. Refinement criteria included: (1) bur‐tip clearance (1 mm safety margin), (2) guide stability during reduction (no wobbling), and (3) tactile feedback clarity. Final validation required 100% accuracy and repeatability in silicone putty stent fits across five trial preparations. (Figure 1)

**FIGURE 1:**
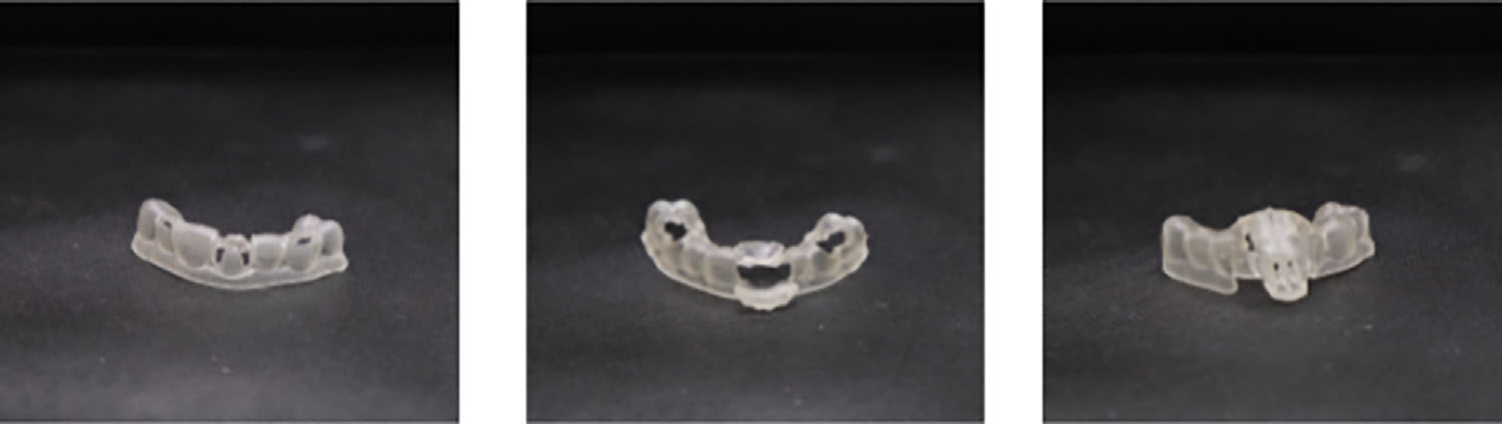
3D‐printed reduction guides (left to right: Steps 1–3) and concise digital workflow (scan → CAD design → segmentation → 3D print → post‐processing → seating).

### 2.2 Preclinical Study Design and Participants

This preclinical comparative study involved 25 third‐ and fourth‐year dental students. We recruited D3–D4 students because crown preparation is taught in Year 2 at our school, so participants had completed the preclinical coursework and were experienced with simulated preparations but had performed fewer than five clinical preparations; therefore, we describe them as experienced in preclinical tooth preparation but early in their clinical learning. Each participant completed two full‐crown preparations on a maxillary typodont incisor (tooth #8) during a single laboratory session—one preparation using the segmented tri‐component reduction guide (guided condition) and one preparation performed free‐hand (control condition). (Figure 2) All preparations were performed with the typodonts mounted in standard manikin simulators. Immediately prior to data collection, every student received an identical standardized pre‐brief that reviewed the CDCA/ADEX all‐ceramic anterior crown preparation standards (including target axial and occlusal reductions, margin configuration, and taper) and included a demonstration of how to use the 3D‐printed reduction guide. Participants completed a short supervised practice using the guide to become familiar with seating and handling, but no hands‐on coaching was provided during the timed preparation tasks. To control for order effects, each student completed both conditions sequentially within a single laboratory session in randomized order. No formal washout interval was imposed; transitions were participant‐paced. Standard high‐speed instrumentation and diamond burs (6856‐016, 6856‐021, 8856‐016, 6878K‐012, 6379‐023, 8847KR‐018, 6847KR‐018, 10839‐014) were used.

**FIGURE 2:**
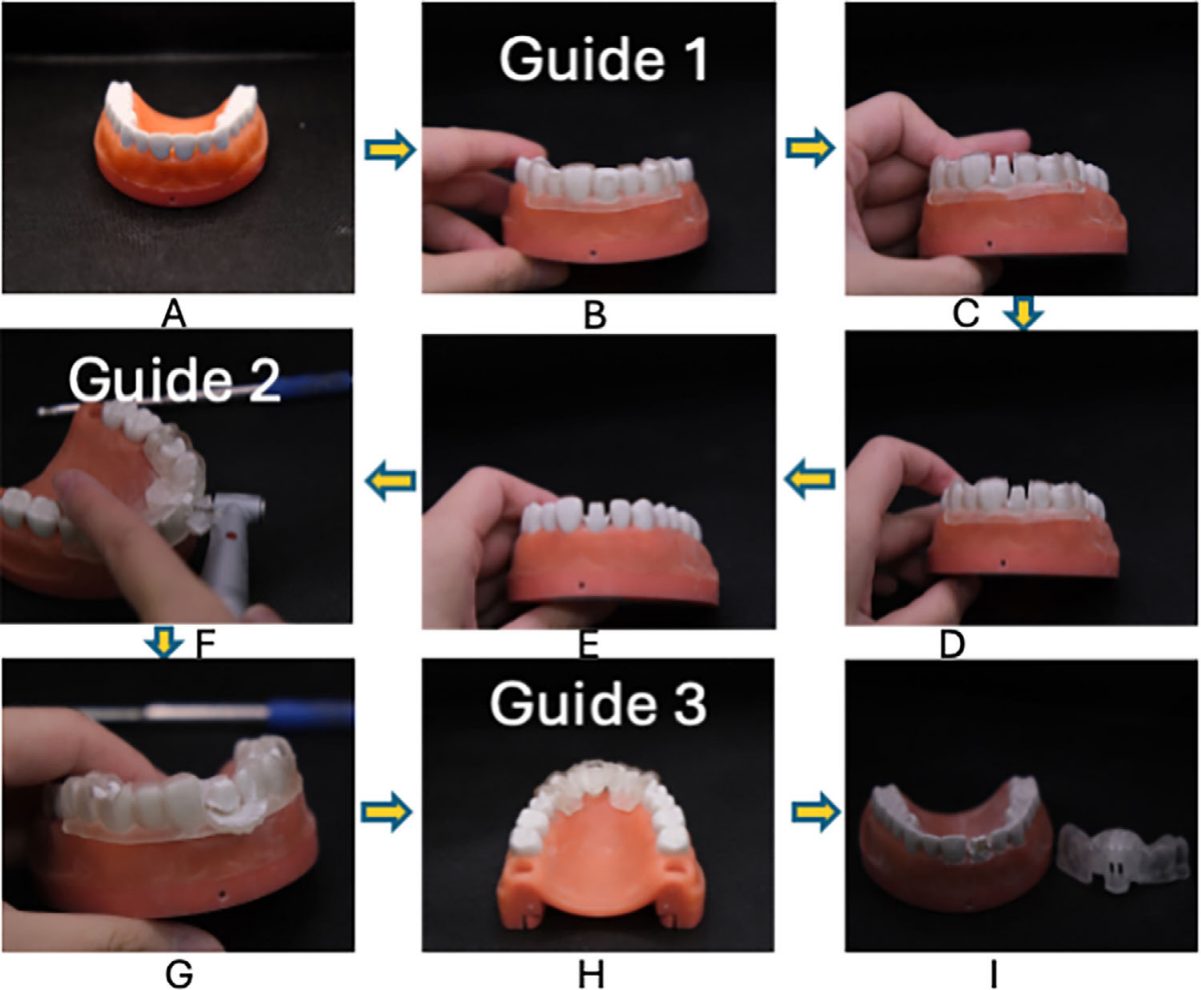
Application of the 3D‐printed guides in three sequential cutting stages on the typodont incisor: Guide 1 (initial incisal/axial reduction), Guide 2 (labial/lingual marginal ridge reduction), Guide 3 (final facial/lingual reduction and finishing). Yellow arrows indicate the stage progression.

For the guided preparation, the student sequentially applied the 3D printed guides to tooth #8: after completing the cuts permitted by the first‐stage guide, it was removed and replaced with the second‐stage guide, followed by the third‐stage guide. This stepwise approach directed the reduction of tooth structure in stages according to the guide design. For free‐hand preparation, students followed the conventional approach without any guiding device, relying on their judgment to achieve the target preparation form.

### 2.3 Outcome Assessment and Timing

Upon completing each preparation, the outcome was evaluated using both digital and manual assessment techniques. Prepared teeth were scanned and analyzed in CEREC SW 5.2 with PrepCheck (Dentsply Sirona, Charlotte, NC, USA) against an ideal model; software‐derived measurements included incisal reduction, axial taper, and margin width. Two examiners—a prosthodontic faculty member (primary examiner) and a trained senior dental student who assists in preclinical fixed prosthodontics—were blinded to group assignment. Both examiners completed a calibration session prior to data collection. The student examiner functioned as a calibrated secondary rater under faculty oversight. Each scored all preparations independently. Disagreements were discussed to reach a shared interpretation, and when consensus could not be reached, the faculty score served as the final determination. Using silicone putty stents as a reference for overall reduction depth and gross form, along with direct visual inspection, the examiners assessed three additional manual criteria: margin continuity/definition, presence of undercuts, and surface/contour irregularities not readily captured by software analysis. Each of the 11 total criteria was scored according to the ADEX grading rubric as acceptable (ACC), substandard (SUB), or deficient (DEF), corresponding to 0, −1, and −2 points, respectively. The ADEX rubric served as the primary outcome standard; PrepCheck/CEREC‐derived digital metrics were used as adjunctive secondary measures for descriptive/confirmatory analysis. An overall quality score was calculated as the sum across all 11 criteria (range 0 to −22; equal weights).

In addition, the time taken to complete each preparation was recorded in seconds. Timing was standardized. The investigator provided a standardized start cue, and participants started the timing and stopped their own timer when they believed the preparation met the intended form. The investigator watched the display, recorded the time, and resolved any discrepancies on the spot.

All participants completed the postexperience survey immediately after finishing both the guided and free‐hand preparations, before any grading to assess subjective workload. Survey responses were recorded on electronic forms and were not accessible to participants after submission; therefore, students did not have the opportunity to view or modify their responses following receipt of grades. This procedure ensured that subjective workload ratings and perceptions were not influenced by knowledge of examiner scores. The survey included 20 items addressing mental demand, physical demand, perceived performance, effort, and frustration. NASA‐TLX scores were interpreted using the following ranges: *Low* (0–9), *Medium* (10–29), *Somewhat High* (30–49), *High* (50–79), *Very High* (80–100); a global score < 50 indicated “easy to use” [9, 10]. Completed surveys were not accessible after submission. The full survey is provided in Supporting Information (Appendix A).

### 2.4 Statistical Analysis

Power calculations used a priori planning distributions—free‐hand ≈60% acceptable/30% under‐reduced/10% over‐reduced and guided ≈90%/5%/5%—to represent a clinically meaningful improvement; these values informed sample‐size estimation only [11]. Paired tests provided 80% power at *α* = 0.05 for *n* = 25.

The primary outcome was the ordinal quality score (−2 DEF, −1 SUB, 0 ACC), analyzed numerically as paired observations (guided vs. free‐hand) using a paired *t*‐test after verifying approximate normality of differences. The null hypothesis for this comparison was that the mean difference in quality scores between the guided and free‐and methods is zero, versus the two‐sided alternative of a non‐zero mean difference. The secondary outcome was preparation time (seconds), analyzed with paired *t*‐tests. Two‐tailed *α* = 0.05.

## 3 Result

### 3.1 Accuracy and Efficiency (Guided vs. Free‐Hand)

A paired difference analysis evaluated changes in quality scores (guided−free‐hand). Sixteen students (64%) improved, six (24%) showed no change, three (12%) declined by one point, no student declined by > 1 point. (Figure 3). These results suggest that the reduction guide significantly enhanced the accuracy of crown preparations for the majority of students.

**FIGURE 3:**
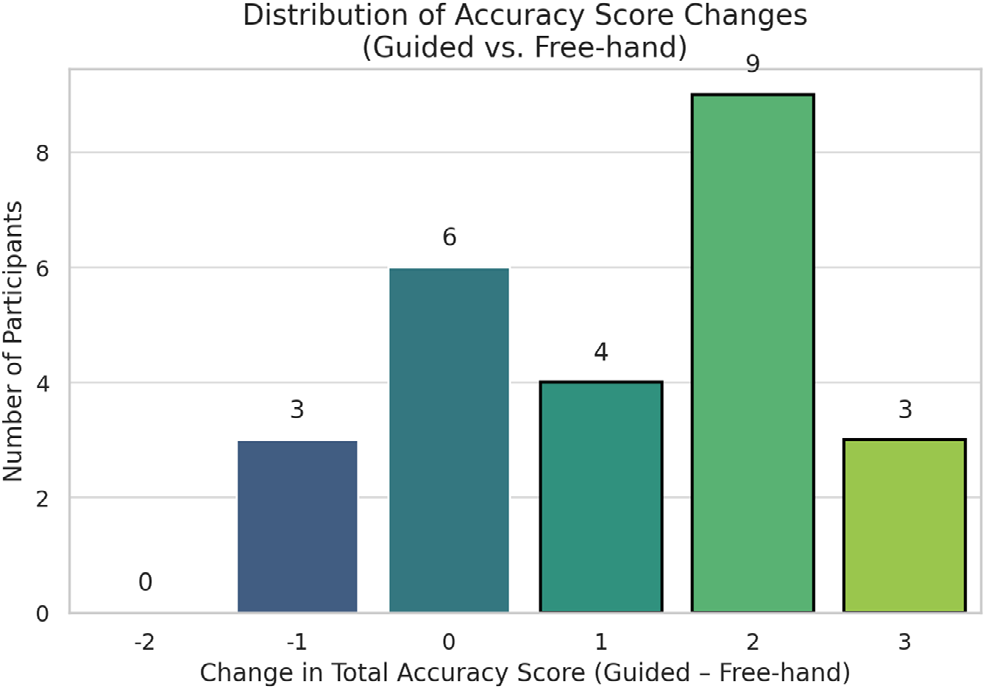
Distribution of preparation quality score changes (guided vs. free‐hand).

A paired *t*‐test showed a statistically significant improvement of crown preparation accuracy with the guide (mean difference 1.12, SD 1.27; *t*(24) = 4.41; *p* = 0.00018). The paired difference plot illustrates that the majority of participants experienced higher preparation quality scores with the guided method compared to free‐hand preparations. The mean improvement of 1.12 points represents approximately 5% of the 22‐point rubric range. Although modest in absolute magnitude, 64% of participants showed improvement, and no student declined by more than 1 point.

Using reduction guides significantly improved preparation accuracy (*p* < 0.05) compared to free‐hand crown preparation, with a 56% increase with guide. Ninety‐two percent of participants had NASA‐TLX < 50; 68% expressed a positive attitude towards using the guide in future practice, while 12% were neutral.

To assess time efficiency, a paired *t*‐test compared mean preparation times. (Figure 4). Free‐hand averaged 30.3 ± 10.6 min; guided averaged 25.2 ± 6.1 min, a significant reduction (*t*(24) = 2.25; *p* = 0.034) corresponding to mean time savings of 5.1 ± 11.4 min.(Table 1)

**FIGURE 4:**
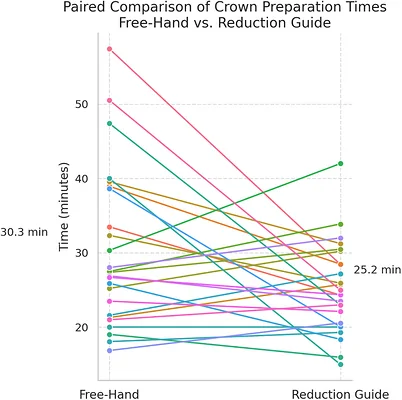
Paired comparison of preparation times reduction guide versus free‐hand.

**TABLE 1:** Paired preparation times for free‐hand and guided conditions.

Participants Group 1	Time wo guide (with 1st, wo 2nd)	Time w guide (with 1st, wo 2nd)
A	21 min 18 s	25 min 44 s
B	32 min 21 s	25 min 55 s
C	25 min 13 s	30 min 13 s
D	27 min 22 s	30 min 29 s
E	30 min 20 s	42 min
F	20 min	20 min
G	18 min 4 s	19 min 18 s
H	21 min 36 s	27 min 12 s
I	25 min 54 s	18 min 22 s
J	38 min 39 s	20 min 4 s
K	16 min 52 s	20 min 34 s
L	28 min 1 s	32 min

Participants Group 2	Time wo guide (wo 1st, w 2nd)	Time w guide (wo 1st, w 2nd)
M	57 min 26 s	28 min 27 s
N	33 min 30 s	24 min 19 s
O	38 min 57 s	28 min 29 s
P	39 min 34 s	31 min 15 s
Q	27 min 30 s	33 min 53 s
R	19 min 02 s	15 min 57 s
S	47 min 24s	23 min
T	40 min	15 min
U	26 min 54 s	23 min 31 s
V	26 min 42 s	24 min 22 s
W	23 min 30 s	22 min 7 s
X	21 min	23 min
Y	50 min 31 s	25 min

It is important to note that students often complete their second crown preparation more efficiently due to the experience gained from the first attempt. Across participants, guided preparations were generally completed more quickly than free‐hand preparations, with a mean reduction of 5.1 min.

### 3.2 NASA‐TLX Survey Result

NASA‐TLX survey responses comparing perceived workload during the learning and use of the 3D‐printed reduction guide. While most students reported low to medium difficulty learning to use the guide, a shift toward higher perceived effort was observed during actual use, with nearly half of respondents rating usage difficulty as “Somewhat High.” (Figure 5)

**FIGURE 5:**
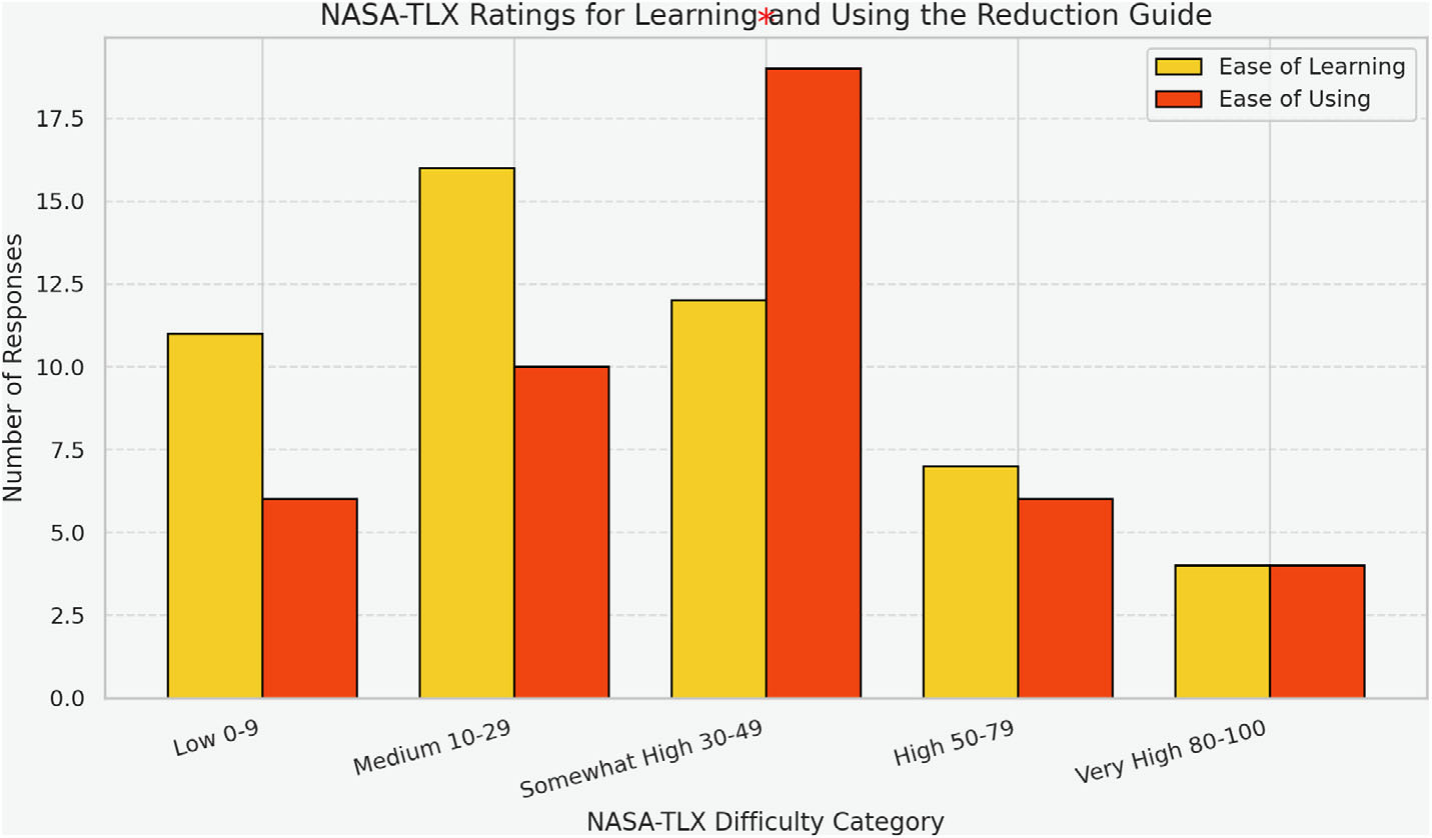
Modified NASA‐TLX survey instrument (Appendix 5).

To evaluate students’ perceived workload associated with learning and using the 3D‐printed reduction guide, a modified NASA Task Load Index (NASA‐TLX) survey was administered. Participants categorized their experiences into five levels of difficulty: *Low* (0–9), *Medium* (10–29), *Somewhat High* (30–49), *High* (50–79), and *Very High* (80–100).

For learning to use the guide, most participants reported relatively low perceived effort: 68% selected Low or Medium difficulty. Only 11% rated the learning process as High or Very High in difficulty. In contrast, the use of the guide during actual preparation was perceived as moderately more demanding. The most frequently selected category was *Somewhat High* (30–49), chosen by 48% of students, while only 40% rated usage difficulty as Low or Medium.

These findings suggest that while the guide is generally easy to learn, its clinical application requires a moderate level of cognitive and physical effort, reflecting the complexity of real‐time crown preparation tasks.

### 3.3 Student Perceptions and Feedback

Students completed a series of Likert‐scale questions to evaluate the perceived impact of the 3D‐printed reduction guide on their crown preparation performance, efficiency, and interest in future clinical applications.

When asked whether the reduction guide improved the accuracy of their crown preparations compared to conventional free‐hand methods (Q15), the majority of students selected scores in the moderate to high range. Specifically, six students rated the accuracy benefit as a “6” and another six selected “7” on a 0–10 scale, indicating a positive perception of the guide's ability to support precise tooth reduction.

Regarding overall effectiveness (Q17), students were asked to rate how helpful the guide was in preparing the crown. Scores ranged primarily from 4 to 8, with the most common rating being “7” (*n* = 8), followed by “5,” “6,” and “8” (each selected by 4 participants). These findings suggest that students generally viewed the guide as a beneficial tool for clinical skill execution.

When asked how likely they would be to use a reduction guide in clinical practice if it were readily available (Q18), responses were diverse but centered around moderate scores, with peaks at ratings of “3,” “7,” and “8.” This distribution indicates that while students recognized the potential value of the guide, some hesitancy remains regarding its immediate adoption into daily practice.

However, after having used the guide in a simulated setting, many students expressed greater interest in applying it to future prosthodontic procedures such as crown, bridge, or veneer preparation (Q19). Interest ratings were highest at scores “6” and “7” (*n* = 5 each), followed by “9” (*n* = 4). These results suggest that firsthand experience with the guide may increase students’ willingness to integrate such tools into their future clinical workflows.

Open‐ended feedback was collected from participants to assess their perceptions of the reduction guide and the overall testing experience. The majority of students responded positively, describing the guide as “a good learning concept” and “a good experience,” suggesting its perceived educational value. However, open‐ended feedback yielded several constructive suggestions, including refining the margin and lingual components and accounting for material removed during finishing/polishing.

## 4 Discussion

This study demonstrated that custom 3D‐printed reduction guides improved anterior crown preparation quality compared to free‐hand methods. The guide was associated with a modest but statistically significant improvement in total preparation quality scores in this simulated setting. Notably, the guides enhanced preparation quality without extending procedural time and significantly reduced total working time by approximately 6.6 min (a 21% decrease). Although some time savings could be attributed to increased familiarity gained from completing multiple preparations, the significant reduction observed under randomized conditions strongly suggests the guides themselves substantially decreased cognitive load. By simplifying decision‐making related to reduction depth, students could direct more attention toward precise technical execution. This interpretation is supported by survey responses indicating a manageable learning curve but a notable reduction in the mental effort required during the guided preparation. Because our findings are limited to a controlled simulation environment with early learners, no conclusions can be drawn about curricular impact or clinical performance. However, for programs interested in studying how digital reduction aids might be evaluated in educational settings, future research could explore a phased approach. Such work might examine: (1) development of an automated software module for guide design, (2) initial training for faculty and students on digital design software, and (3) full incorporation into preclinical curriculum. These points are presented only as conceptual directions for future investigation, not as recommendations for implementation. Additional research—including assessments of learner dependence, transition to free‐hand techniques, workflow burden, and clinical feasibility—would be essential before considering any educational use.

Our findings align well with existing literature, suggesting novice users particularly benefit from visual and tactile reduction aids. Previous research has identified substantial challenges in teaching consistent axial and occlusal reduction through conventional teaching methods, leading to considerable variability in student performance [1, 2]. The standardized form of the guide used in our study notably minimized these variations, helping students consistently achieve preparation standards outlined by ADEX guidelines. The segmented guide design—separating facial, incisal, and lingual reductions—likely further contributed by simplifying procedural steps and allowing focused attention on one geometric plane at a time. Many programs use sectioned PVS putty or vacuum‐formed matrices as analog reduction guides for novice students; they are rapid and inexpensive chairside but provide limited quantitative calibration compared with parameterized digital stop‐planes.

Student feedback supported the educational value of the guides. Participants generally reported ease of use and expressed interest in applying similar tools to future prosthodontic tasks. However, detailed feedback highlighted specific limitations, particularly regarding the margin and lingual aspects of the guide, which students found less intuitive or more challenging to use effectively. These insights identify areas for refinement, particularly visibility, stability, and alignment with typodont anatomy.

Although the guides improved performance in this simulation, their use also raises instructional concerns. Reliance on guided reduction may limit the development of independent evaluative skills, so any educational application would require a clear transition to unguided preparation. Producing patient‐specific guides additionally requires scanning, design, and printing steps that may be impractical in many predoctoral settings. These considerations reinforce that our findings should not be interpreted as supporting curricular use but instead serve as preliminary data for future study.

Despite recognizing the benefits in accuracy and efficiency, students expressed caution regarding the adoption of these guides in clinical practice. This hesitancy likely reflects uncertainty around integrating the technology into existing clinical workflows, concerns about time investment, and questions about the relevance of simulated conditions to real‐patient scenarios. However, increased interest in using guides for prosthodontic cases following hands‐on experience suggests practical exposure could positively influence acceptance.

As intraoral scanning becomes routine for occlusal guards, implant workflows, and crown fabrication, scanned records can be leveraged to design patient‐specific segmented reduction guides using existing CAD tools and printed chairside or via a local laboratory. A feasible clinical workflow would include: (1) acquiring a diagnostic intraoral scan, (2) designing the tri‐component guide referencing planned restorative contours, (3) printing the guide in a biocompatible and sterilizable resin with appropriate post‐processing, and (4) seating the guide at the preparation appointment to guide staged tooth reduction. Practical considerations for adoption include material selection (biocompatibility and sterilization compatibility), staff training in digital design and guide seating, cost and turnaround time for in‐house versus outsourced printing, and infection‐control policies (single‐use vs. sterilizable guides). Clinics should incorporate guide files into the patient's digital record to support treatment continuity. Prospective clinical studies are needed to confirm that the benefits observed in typodont simulations translate to real‐patient outcomes and cost‐effectiveness in routine practice.

This study's strengths include its robust within‐subject design, objective digital measurements, and validated workload assessments. Nevertheless, it is important to acknowledge limitations such as the relatively small sample size, the artificial nature of typodont preparations, and the exclusive focus on anterior crowns. In particular, the modest sample (*n* = 25) limits precision for subgroup analyses (e.g., D3 vs. D4) and may have reduced power to detect smaller effects on secondary outcomes (e.g., preparation time or specific NASA‐TLX domains). Additional limitations include the single‐institution, early‐learner cohort (D3–D4 with < 5 prior clinical crowns), which may limit generalizability; a brief presession familiarization with the guide without a contemporaneous free‐hand warm‐up, which could introduce a small familiarity effect. Although examiners were blinded and cases were de‐identified and interleaved, complete masking cannot be guaranteed. However, because finish‐line vertical level was not an ADEX scoring item and scoring packets contained no condition markers, the risk of unmasking was considered low. No standardized washout interval was used between sequential conditions; although order was randomized, carryover effects cannot be fully excluded. Finally, we used CEREC SW 5.2/PrepCheck measurements to supplement the ADEX rubric. These metrics did not replace the rubric, and their values may not be directly comparable with those from other software. This study serves as a guide for next‐phase clinical research: a pragmatic trial in patients comparing guided versus free‐hand anterior crowns with outcomes that include reduction accuracy from intraoral scans, chairside adjustment time, marginal adaptation, postoperative sensitivity, gingival indices adjacent to the crown, and short‐term restoration survival (6–12 months). Multicenter designs and inclusion of posterior teeth will further test generalizability and cost‐effectiveness.

## 5 Conclusion

This preclinical simulation study found that digitally designed, custom 3D‐printed reduction guides improved preparation accuracy and shortened working time for anterior crowns versus free‐hand, confirmed by digital metrics and blinded examiner scoring. Future work should include posterior teeth, larger cohorts, patient cases, and analysis of curricular effects and inter‐rater reliability. In addition, develop a student workflow for designing the guides (scan import, segmentation, and print) and a simple translation method that adapts typodont designs to patients by substituting intraoral scans and re‐registering geometry.

## Disclosure

The authors have nothing to report.

## Conflicts of Interest

The authorShuncong Xu, who contributed to the design of the reduction guide in this study is listed on a provisional patent application related to this guide.

## Supporting information




**Appendix A**: Modified NASA‐TLX Survey Instrument.

**Supplement File 2**: jdd70163‐sup‐0002‐SuppMat.pdf.
