# Supplementary material for: Comparing Digital Reduction Guides and Free‐Hand Techniques for Predoctoral Crown Preparation Accuracy
Source: J Dent Educ. 2026 Feb 10;90(10):1646–52. doi: 10.1002/jdd.70163 (PMC13665596; doi:10.1002/jdd.70163)
Supplement: Supplementary file 1 [file JDD-90-1646-s002.pdf]

CERAMIC CROWN PREPARATION

ADEX 2024

CRITICAL ERRORS		
Wrong tooth/surface treated	No	Yes
Procedure not challenged	No	Yes

NOTE: Those SUBs that are highlighted are part of the 3-SUB Rule

ACC = Adheres to Criteria SUB = Marginally Substandard DEF = Critical Deficiency

CERVICAL MARGIN AND DRAW	
Margin/Extension	
ACC	The cervical margin is ≤ 0.5 mm below the simulated free gingival margin to ≤ 1.5 mm incisal to the simulated free gingival margin.
SUB	A. The cervical margin is over-extended > 0.5 mm below the crest of the simulated free gingival margin.
DEF	A. The cervical margin is over-extended > 0.5 mm below the simulated free gingival margin, causing visual damage to the typodont.<br>B. The cervical margin is under-extended by > 1.5 mm above the simulated free gingival margin.
Margin/Definition/Unbeveled	
ACC	The cervical margin is continuous but may be slightly rough and may lack some definition.
DEF	A. The cervical margin has no continuity and/or definition.<br>B. The margin is beveled.<br>C. The margin is cupped or J-shaped.
Margin/Cervical Width	
ACC	The cervical margin width is ≥ 0.5 mm but ≤ 1.5 mm in width.
SUB	A. The cervical margin width is > 1.5 mm but ≤ 2.0 mm.
DEF	A. The cervical margin width is > 2.0 mm in width.<br>B. The cervical margin width is < 0.5 mm.
Line of Draw	
ACC	The path of insertion/line of draw deviates < 20° from the long axis of the tooth.
SUB	The path of insertion/line of draw deviates 20° to < 30° from the long axis of the tooth.
DEF	The path of insertion/line of draw deviates ≥ 30° from the long axis of the tooth.
WALLS, TAPER, AND MARGIN	
Axial/Lingual Tissue Reduction	
ACC	The axial/lingual tissue reduction is ≥ 1.0 mm but ≤ 2.0 mm.
SUB	A. The axial/lingual tissue reduction is > 2.0 mm but ≤ 2.5 mm.
DEF	A. The axial/lingual tissue reduction is > 2.5 mm.<br>B. The axial/lingual tissue reduction is < 1.0 mm.
Axial Walls Smoothness/Undercut	
ACC	The walls may be slightly rough and may lack some definition.
DEF	There is an undercut, which, when blocked out, would compromise margin width criteria and/or is > 0.5 mm deep.

CERAMIC CROWN PREPARATION (CONTINUED)

**Taper**	
ACC	Taper is present, from nearly parallel to ≤ 12° per wall.
SUB	There is excessive taper that is > 12° but ≤ 16° per wall.
DEF	The taper is grossly over-reduced > 16° per wall.
**Incisal Reduction**	
ACC	The incisal reduction is ≥ 1.0 mm but ≤ 3.0 mm.
SUB	The incisal reduction is > 3.0 mm but ≤ 3.5 mm.
DEF	A. The incisal reduction is > 3.5 mm.<br>B. The incisal reduction is < 1.0 mm.
**External/Internal Line Angles**	
ACC	External and/or internal line angles may be rounded but irregular.
DEF	The external and/or internal line angles are excessively sharp with no evidence of rounding.
**Lingual Wall Height**	
ACC	The lingual wall height is ≥ 1.0 mm.
DEF	The lingual wall height is < 1.0 mm.
**TREATMENT MANAGEMENT**	
**Condition of Adjacent/Opposing Teeth**	
ACC	Any damage to adjacent tooth/teeth can be removed with polishing without adversely altering the shape of the contour and/or contact.
SUB	A. Damage to adjacent tooth/teeth requires recontouring that changes the shape and/or position of the contact.<br>B. Opposing hard tissue shows minimal evidence of damage and/or alteration inconsistent with the procedure.
DEF	A. There is gross damage to adjacent tooth/teeth, requiring a restoration.<br>B. There is evidence of gross damage and/or alteration to opposing hard tissue inconsistent with the procedure.
**Condition of Surrounding Tissue**	
ACC	There may be slight damage to the simulated gingiva and/or typodont consistent with the procedure.
SUB	There is iatrogenic damage to the simulated gingiva and/or typodont inconsistent with the procedure.
DEF	There is gross iatrogenic damage to the simulated gingiva and/or typodont inconsistent with the procedure.
