# Supplementary material for: Comparing Digital Reduction Guides and Free‐Hand Techniques for Predoctoral Crown Preparation Accuracy
Source: J Dent Educ. 2026 Feb 10;90(10):1646–52. doi: 10.1002/jdd.70163 (PMC13665596; doi:10.1002/jdd.70163)
Supplement: Supplementary file 2 [file JDD-90-1646-s001.docx]

Appendix A. Modified NASA-TLX Survey Instrument

This survey was administered following both crown preparation procedures to assess students’ perceived cognitive and physical workload, ease of use, and overall impressions of the 3D-printed reduction guide. Items were rated on a scale from 0 (very low) to 10 (very high) unless otherwise noted.

Section 1: Instruction Clarity

The instructions for using the reduction guide (learning goals) were clear.

The content about how to use the reduction guide was presented in an understandable way.

What aspects of the reduction guide were most difficult to understand or use? (open-ended)

Section 2: Learning to Use the Guide

How mentally challenging was it to learn to use the reduction guides? (Mental Demand)

How physically challenging was it to learn to use the reduction guides? (Physical Demand)

How hurried or rushed was the pace while learning to use the guides? (Temporal Demand)

How successful were you in learning to use the reduction guides? (Performance)

How much effort did you invest in learning to use the guides? (Effort)

How frustrated or stressed did you feel while learning to use the guides? (Frustration)

Section 3: Using the Guide During Preparation

How mentally challenging was it to use the guides for crown preparation? (Mental Demand)

How physically challenging was it to use the guides compared to the free-hand technique? (Physical Demand)

How rushed did the process feel when using the guides versus free-hand? (Temporal Demand)

How successful were you in completing the preparation using the guides? (Performance)

How much effort did using the guide require compared to free-hand preparation? (Effort)

How frustrated or stressed did you feel while using the guide? (Frustration)

Section 4: Perception of Guide Effectiveness

Did the reduction guide improve the accuracy of your preparation compared to free-hand? (Yes/No/Explain)

Approximately how much time did you save using the guide vs. free-hand? (in minutes)

On a scale of 1 to 10, how effective was the guide in helping you prepare the crown?

If reduction guides were available in your clinical training, how likely would you be to use them? (1 = not at all likely, 10 = very likely)

Has your interest in using digital guides for future cases (crowns, bridges, veneers) increased after this experience? (Yes/No)
